# Visual Analysis of Research Paper Collections Using Normalized Relative Compression

**DOI:** 10.3390/e21060612

**Published:** 2019-06-21

**Authors:** Pere-Pau Vázquez

**Affiliations:** ViRVIG Group, Universitat Politècnica de Catalunya, 08034 Barcelona, Spain; pere.pau@cs.upc.edu

**Keywords:** visualization, compression, similarity, text

## Abstract

The analysis of research paper collections is an interesting topic that can give insights on whether a research area is stalled in the same problems, or there is a great amount of novelty every year. Previous research has addressed similar tasks by the analysis of keywords or reference lists, with different degrees of human intervention. In this paper, we demonstrate how, with the use of Normalized Relative Compression, together with a set of automated data-processing tasks, we can successfully visually compare research articles and document collections. We also achieve very similar results with Normalized Conditional Compression that can be applied with a regular compressor. With our approach, we can group papers of different disciplines, analyze how a conference evolves throughout the different editions, or how the profile of a researcher changes through the time. We provide a set of tests that validate our technique, and show that it behaves better for these tasks than other techniques previously proposed.

## 1. Introduction

Visualizing the evolution of a certain researcher or conference, getting insights on whether it is evolving or stalled, is an interesting problem that has been addressed previously through the analysis of co-occurrence of topics. However, to the authors’ knowledge, most of the previous analysis requires a big amount of human effort in identifying, refining, and classifying keywords or topics [1]. Moreover, obtaining visualizations that communicate at a glance how a full conference or the research of an author has evolved throughout the years is challenging. In this paper, we propose some techniques for the fully automated analysis of research papers with the objective of visually presenting information on how the contents of the research papers of an author or a conference have changed. This may increase the understanding of the profile of an author and give clues on the innovative quality of her research. We propose the use of information theory-based measures, such as the Normalized Relative Compression, NRC (and also the Normalized Conditional Compression, NCC), to compare papers. With it, we can estimate the amount of a paper that can be explained by another. Though NRC is not a distance, e.g., is not symmetric, and does not fulfill the triangle inequality, it still seems a good predictor on the *innovative nature*, or the difference of one article versus another. We believe that the difference measure used throughout this paper (that measures the proportion of a string that cannot be computed with the other), gives an estimate of the innovation, or the degree upon which a text presents novel techniques or addresses problems not dealt with in another paper. We use NRC to compare single papers as well as documents collections, and provide visualizations to communicate the evolution of a full conference edition by comparing it with other editions. Moreover, we also provide fine grain insights by visualizing the measured differences between all the papers of one edition versus all the papers of the other.

To sum up, the contributions of this paper are:

- A method for comparing the relative difference of a paper with respect to another.
- A fully automatic pipeline to process and evaluate PDF files of research articles.
- A series of visualizations intended to illustrate conference or author profile evolution.

As already stated, we propose the use of NRC for paper comparison, but instead of using it in plain files, we will perform a series of text processing tasks, borrowed from machine learning, to improve the comparison results. These tasks will be applied in a totally automated fashion using a series of scripts and Python programs. To ensure that the measure behaves as expected, we have run a set of tests that show that NRC, with the processed data, seems a good estimator of paper difference. Finally, we propose some visual depictions to communicate this information for full conference editions, author profiles, and the whole set of documents of a conference.

The rest of the paper is organized as follows. First, we will analyze previous work. Then, the selected corpus of papers is presented, and an initial analysis is carried out using word frequencies in Section 3. Next, we propose and validate a pipeline for the analysis of research papers in Section 4 using Normalized Relative Compression. In Section 5 we present the visual depictions proposed for illustrating conference or author research evolution. Finally, Section 6 summarizes the results and concludes our work.

## 2. Previous Work

Text analysis tools and techniques have been previously developed with several objectives such as spam identification, plagiarism detection, authorship attribution, and so on. In visualization, it has been used to visually classify text documents, and to analyze the evolution or research papers [1]. Our paper borrows tools of text analysis and comparison to visualize the evolution of research. Therefore, we will review some previously used techniques to evaluate text, and then the papers more related to our goal: the visual communication of research evolution.

### 2.1. Text Analysis

Text analysis comes in very different flavors that depend on the tasks to be performed [2]. For example, a lot of effort is being put into finding vector representations for unstructured text, because the Internet grows exponentially, and large amounts of data are created every day. Deep learning techniques typically address this by transforming texts onto vector representations [3], to address several tasks such as document clustering or data mining [4,5]. The popular Bag-of-Words family of methods maps a document to a fixed-length vector. Typically, vector representations (also known as word embeddings) can be used to determine distances between words. However, the use of Bag-of-Words for direct document comparison is problematic because it does not contain the notion of similarity between words. Thus, sentences that may be synonymous may pass undetected (Kusner et al. [4]). Although some developments try to circumvent this problem, such as the Latent Semantic Indexing or Latent Dirichlet Allocation, they commonly do not improve over BOW in distance-based problems such as the one addressed in this paper. One extension that addresses document distance estimation is Word Mover’s Distance (WMD). WMD is a metric by Kusner et al. [4,6] whose goal is to measure distances between documents, even when they have no words in common. WMD is inspired on the Earth Mover’s Distance (EMD) [7], which basically turns the similarity measurement into a transportation problem, and it measures how difficult is it to *move* from one representation to another. The metric has been used in many applications, such as image similarity comparison [8,9]. Kusner et al. [4] designed their own version of the EMD by starting from an embedding representation generated using the algorithm *word2vec*.

Another popular family of methods to compare texts is the use of compression-based measures. Compression-based techniques usually require less effort than deep learning approaches, and sometimes can be parameter-free. Pinho et al. use Normalized Relative Compression for authorship attribution of texts [10] and report a higher success ratio than with another popular measure, the Normalized Compression Distance [11]. Cerra and Datcu develop a similar measure, called Normalized Relative Complexity, based on an approximation of the Kolmogorov cross-complexity, which leads to a formulation that is slightly different than the NRC proposed by Pinho and colleagues. They later extend the technique for pattern recognition [12]. Cerra et al. also deal with the problem of text processing using a measure called Fast Compression Distance, that that computes the similarity between two texts using the intersection set between two dictionaries. Though this is faster than typical compression, their success rates seem slightly below the ones reported by Pinho et al. [10]. Oliveira et al. [13] compare the performance of some compression-based measures. Helmer [14] uses entropy-based and Kolmogorov-based measures for structural similarity detection of semistructured documents. Coutinho and Figueiredo [15,16] addresses the problem of text sentiment analysis and text classification, also using information-theoretic measurements. More concretely, they use relative entropy estimation between pairs of sequences of symbols, and then use classical classifiers (e.g., support vector machines) for text classification. Other applications of compression-based measures have been demonstrated by Pratas and colleagues, for genome analysis [17,18] or Carvalho et al. for electro-cardiogram classification [19].

### 2.2. Visualization of Research Papers

In the area of visualization, the problem of visually communicating information relative to the research paper has also been addressed. Isenberg et al. recently analyzed keywords of scientific papers to explore the evolution of IEEE Vis conferences [1]. In this paper, the authors performed a manual extraction and classification of keywords in scientific papers. The posterior analysis of the co-occurrence of such words serves to get an approximation of the problems that have been most frequently addressed in visualization. As a result, the authors also published a database of IEEE VisWeek data [20]. Moreover, the authors have also created a web application that can be used to query [21] those keywords and gain knowledge of the papers that use a set of keywords. Similar analysis has also been carried out in other areas such as Human Computer Interaction, software engineering and others [22,23,24,25,26]. Our work shares this spirit, but with the idea of providing information on how a certain conference or author profile has evolved. Therefore, we seek information on whether a certain research area changes with time. In addition, we do so by analyzing the actual texts of the papers themselves, and creating a fully automated pipeline for paper comparison.

Some previous techniques are also related to our work here, such as the systems that analyze the texts to perform different tasks such as literature recommendation. Ponsard et al. [27] developed Paperquest, a system that uses text analysis to aid in the selection of appropriate literature, by generating sets of papers that are relevant to users according to information on their interest, and the number of citations. A similar approach, due to Nikhil and Srivastava, uses deep learning to recommend documents based on similarity [28]. In this case, the authors use *word2vec* distributed representations of words [29], coupled with a Convolutional Deep Structured Semantic Model (CDSSM) [30]. In our case, we also compare our paper similarity technique with a deep learning-based technique (Word Mover’s Distance) and show that the measure we employed seems to be superior for our goal.

## 3. Initial Analysis

Before delving into the more complex domain of text comparison, we approached the problem using a naive analysis technique: studying words frequencies. The idea is to explore how far this strategy can go, and what are the difficulties with the input data. To begin with the analysis, we need to select a suitable set of papers to analyze, as described next.

### 3.1. Data Selection

To demonstrate the different algorithms, we decided to work with visualization papers. Consequently, we use all the full papers of the IEEE VisWeek for the last 10 years. There are several reasons behind this paper selection. First, the total number of papers in a regular VisWeek from the three main conferences is larger than 100, which makes human processing of the data very difficult. Second, we want to keep the input data as homogeneous as possible, so this may be a suitable data source. To preserve homogeneity, we decided to use only the papers that were published by the TVCG journal, which amount a total of 999 for these years. Although VAST as a conference exists since 2006, it was not incorporated to the VisWeek until year 2011. However, the papers did not appear in the TVCG journal until 2012. Using the same format for the input data is also important because it guarantees that the preprocessing we are applying to the data will have the same results. To further keep the consistency of the format, the input data was downloaded as PDFs from the IEEEXplore website.

### 3.2. Word Frequency Analysis

**Figure 1:**
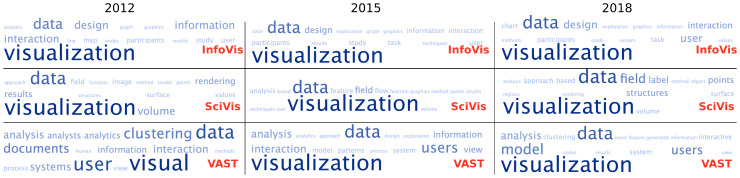
A simple analysis of most frequent words of the InfoVis, SciVis, and VAST conferences of the IEEE VisWeek for the years 2012, 2015, and 2018.

The simplest way to reduce a text to some small meaningful representation is the selection of most repeated words. Though with its own limitations, word frequencies can somewhat communicate the general topics discussed in a document. Most frequent words can be displayed in an intuitive way by using word clouds. As a first attempt, we will use such clouds to illustrate the evolution of the three main VisWeek conferences by comparing three years, namely 2012, 2015, and 2018. In our example, we select the 15 most frequently used words, and depict them in Figure 1. To facilitate reading, we use a word cloud implementation where all words are displayed horizontally, and the frequency is denoted by size and opacity. Here the first obstacle appears: some words that appear frequently have little to do with the contents of the paper, such as “using”, “used”, “computer”, or “different”. For this initial test, these have been avoided. Thus, if we keep with the top 15 most frequently written words the different editions of the VisWeek conferences, we end up with Figure 1.

If we inspect these most frequent words, we find some expected results, such as the words “data” or “visualization” among the highest frequencies. A deeper analysis lets us see that SciVis seems to contain a larger word set than the other conferences, i.e., there is more variety in the most mentioned words.

Unfortunately, reducing the conference to a small set of words is extremely lossy, and therefore, these reduced samples may underrepresent the big picture. A further step could consist of a more thorough analysis of the most frequent topics per year, or analyzing keywords, as in [1]. While topics analysis per year would paint a more detailed picture, it requires a human effort to discard non-interesting words. Isenberg et al. [1] found that extra work was required to properly classify keywords, since different authors might use different words to relate to the same topic. Since one of our goals is to keep the data processing simple, and as much automatic as possible, we cannot follow this path.

On the other hand, this initial analysis sheds light on the need for properly dealing with the input data to ensure its quality and robustness. Therefore, the initial steps before comparing texts will use automated techniques erase stop words, as well as properly cleaning the files of extraneous symbols.

We also learnt another property of the data: the same word (or concept) may be written differently, e.g., use or using will mean something similar, but if they are counted as different words, measures based on frequency will not work. To solve this, we will use another well-known technique from natural language processing, word stemming, as will be explained later.

## 4. Similarity Measurement Using NRC

### 4.1. Requirements

We had two main goals when addressing this project. We wanted to visually depict the evolution of a conference as a whole, and we also wanted to obtain visual descriptions of the evolution of a certain author profile. To do this, previous methods for research analysis fall short. Therefore, we seek a measure of paper difference with the following properties:

- Indicates the degree of novelty of a certain paper.
- Can be used to classify papers by research areas.
- Is able to further separate within papers of the same area.

These properties are difficult to express formally. Even an expert might find difficult to measure the *degree of novelty* of a paper. Consequently, it is not straightforward to find a baseline to use as reference for all these properties. Therefore, as explained below, we created a series of synthetic tests that cover all desired cases. For example, we can compare the degree of novelty by analyzing a paper and a follow-up of the same paper. Or we can easily determine whether visualization papers can be separated from other areas by comparing them with several papers of different fields downloaded from *arxiv.org*, and so on.

### 4.2. Data Preparation

This process includes gathering the files and processing them. For the comparison of full conferences, we downloaded all the papers of the IEEE VisWeek conferences for the last 10 years. In this case, the input data consists of the PDFs downloaded from the IEEEXplore website. For the individual tests for validation, we use a mix of papers obtained from these conferences, as well as other downloaded from either *arxiv.org* or from some author web pages. These extra documents are enumerated in the Appendix A and Appendix B.

To perform a systematic analysis of data, the treatment must be the same. With the lessons learnt from the word frequency analysis, we define a processing pipeline that consists of three main steps, applied to each paper:

1. Text extraction.
2. Data cleaning.
3. Word stemming.

The *text extraction* can be done in different ways, but finally opted for the *pdftotext* software. We initially tried with different tools, but the results were never perfect, and this one seemed to have the most consistent behavior. In some rare cases, the generated files consisted on binary files or contained a lot of garbage, thus making them unusable. In those cases, we also tested Adobe software for the same articles, but it also had problems for generating the text. Consequently, we had to remove half a dozen of files for the comparison tasks. This is a limitation that can be addressed in the future, as discussed later. The second step is *data cleaning*. This was carried out by using a series of Python and Perl scripts that perform the following tasks:

- Stopword removal.
- Removing spaces and changing text to lowercase.
- Erasing non-printable characters.
- Removing extraneous characters.

*Stopwords* are words that are very common in English, such as ’we’, ’our’, and so on, but that do not change the semantics of a text significantly. Learning approaches remove stopwords as part as the data cleaning. Extra spacings are also non-significant, and are thus also removed. Besides, to facilitate word comparisons, learning techniques also change the letters to lowercase. We also do this by a Python script with the *nltk* library. The extracted text often contains extraneous characters such as many points or non-printable characters. A couple of Perl scripts take care of those.

We considered and tested other potential text processing, such as removing words that are non-existent in English, or removing all words with letters not belonging to the alphabet, but all of them have shortcomings. For example, erasing words not belonging to the English language would eliminate most authors’ names. Since highly repeated names may indicate similarities (e.g., papers that have similar reference lists might deal with a similar technique or problem) we would somewhat hurt the similarity search. Moreover, sometimes authors coin a certain name to dub a technique that is then used repeatedly, and might not appear in a regular English dictionary. Removing letters not belonging to the alphabet has also some issues: for instance, it mutilates, if not completely erases all formulae in the papers, years in citations, and other names that are commonly used, such as 3D. Therefore, we have preferred to work with the resulting data of the previous processing. Learning techniques also transform digits into letters, but we preferred to avoid this to preserve (as much as the pdftotext allows) equations.

Finally, we further process the text by using a well-known technique named stemming. Stemming is the process that intends to reduce word inflections to their stems. In our case, we used Porter stemming [31], although other possibilities are also valid alternatives. Again, this processing is carried out in Python using the *nltk* library.

Although the original data requires more than one GB, after text extraction, cleaning, stop word removal, and stemming, the result is a set of files that amounts around 50MB.

### 4.3. Similarity Measures

As already mentioned, there are several ways in the literature to measure similarity between documents. In our case, the fundamental problem when analyzing the contents of the articles is to be able to compare texts. Some popular measures for text comparison are Normalized Compression Distance [11] or Normalized Relative Compression [10]. Normalized Compression Distance (NCD) is a measure based on Kolmogorov complexity that estimates the similarity between two strings by using a *normal* (real-world) compressor. A compressor is *normal* if it fulfills the properties of idempotency, monotonicity, symmetry and distributivity up to a logarithm term [11]. Normalized Compression Distance is measured as:

(1) $$NCD\left(x,y\right)=\frac{C(xy)-min\{C(x),C(y)\}}{max\{C(x),C(y)\}},$$

where function C(f) is the size of the compression of a certain file *f*, and xy is the concatenation of files *x* and *y*. Although the similarity metric has values in [0…1], NCD values are usually in the range of [0…1.1], due to real-world compressor imperfections [11]. NCD has been used for applications, for example language classification and handwriting recognition [11], image similarity evaluation comparison [32,33], or file fragment classification [34], to name a few.

Normalized Relative Compression, on the other hand, is similar to the previous one, but its formulation depends on the use of a conditional compressor. It measures the portion of one string that cannot be computed with the other. It can be calculated using the following expression:

(2) $$NRC\left(x,y\right)=\frac{C(x∥y)}{\left|x\right|},$$

where C(x∥y) represents the compression of *x* relative to *y*. Pinho et al. [10] developed a special compressor that uses finite context models and can perform conditional compression as well as relative compression. Relative compression is achieved with a two-step process. Given a reference file *y*, the compressor builds a representation of it using finite context models of different orders, and then builds a representation of *x* based on this representation. As a result, the relative compression measures how much of the second file can be built using the reference file.

This measure fulfills the following properties:

1. C(x∥y)≈0 iff string *x* can be built efficiently from (is very similar to) *y*;
2. C(x∥y)≈|x| iff C(x|y)≈C(x);

where C(x) means the size of compressed file (an approximation of Kolmogorov complexity using compressors), and C(x|y) is the conditional compression of *x* given *y* (which approximates the conditional Kolmogorov complexity using compressors [11,35]).

NRC has been used for applications such as authorship attribution [10], or studying the evolution of primate genomes [17]. The main advantage of NCD over NRC is that it can be evaluated using a common compressor, while the NRC requires a special-purpose one that is able to perform conditional compression. However, the interesting difference comes from what they measure. NCD measures how two strings (files, images*…*) are different in terms of the contents, while NRC of two strings attempts to answer on *the portion* of the second string that cannot be constructed from information of the first one [17]. With this in mind, it seems that both measures can be used for paper comparison, although the non-symmetry of NRC seems to be useful to determine the degree of novelty of a paper with respect to the other (provided that the differences in both directions vary), since it measures the amount of information that cannot be computed with the other. Another important feature of NRC is that it does not preserve the triangle inequality.

Another similarity measure that has been used to compare texts is the so-called Normalized Conditional Compression Distance [36], which is defined as:

(3) $$NCCD\left(x,y\right)=\frac{max\{C(x|y),C(y|x)\}}{max\{C(x),C(y)\}},$$

where C(x|y) is the *conditional compression* of the sequence *x* given *y*. Conditional compression can be achieved using a special compressor that takes *y* as input and builds an internal model of *y*. In our case, we use the compressor by Pinho et al. [10], which builds this model by using a combination of finite context models (FCMs) of several orders. After processing *y*, *x* is compressed using these previously defined models and another set of FCMs that learn the statistics of *x* as it is processed. Each symbol of *x* is encoded using a probability estimate, resulting from a mixture of the probabilities produced by each of the FCMs (those modeling *y* and those modeling *x*). Conditional compression can also be approximated by C(x|y)=C(yx)-C(y), which can be calculated with a general real-world compressor [37]. However, to calculate NCCD, even with this approximation, requires compressing a sequence (either the individual files or the concatenations) four times. This makes this approach slower than relative compression, as will be detailed later.

A similar measure is the Normalized Conditional Compression, which can be calculated as:

(4) $$NCC\left(x,y\right)=\frac{C(x|y)}{C(x)},$$

where, as in the previous case, C(x|y) is the *conditional compression* of the sequence *x* given *y*. NCC is also a semi-distance, like NRC, and we will also evaluate its performance.

Lately, deep learning techniques have been used for text processing. A popular family of methods are based on word embeddings. One of such techniques is WMD. WMD is a metric by Kusner et al. [4,6] whose goal is to measure distances between documents, even when they have no words in common. WMD is inspired on the Earth Mover’s Distance (EMD) [7], which basically turns the similarity measurement into a transportation problem, and it measures how difficult is it to *move* from one representation to another. The metric has been used in many applications, such as image similarity comparison [8,9]. Kusner et al. [4] designed their own version of the EMD by starting from an embedding representation generated using *word2vec*. Other techniques have been proposed previously, such as the use of Bag-of-Words directly. However, as reported by Kusner et al. [4], it is problematic because it does not contain the notion of similarity between words, so sentences that may be synonymous may pass undetected.

### 4.4. Validation

To ensure that the measures work as intended, it is of uttermost importance to validate the measure we selected with our goal in mind. Therefore, we run different validation tests intended to ensure that NRC is measuring what we expected, and to demonstrate that it works better than other alternatives. One of the most important elements to ensure is whether the formulation is correctly estimating the difference between two files (we call this the *accuracy test*). Then, we need to discard that NRC is effectively measuring author style instead of the contents of the paper, since it has been used for this goal previously in the literature. Then, we need to test whether NRC can separate papers of different areas. Besides, we also need to check the behavior of the technique against other metrics proposed in the literature, such as NCCD, NCC, NCD, and WMD.

#### 4.4.1. Accuracy Test

**Figure 2:**
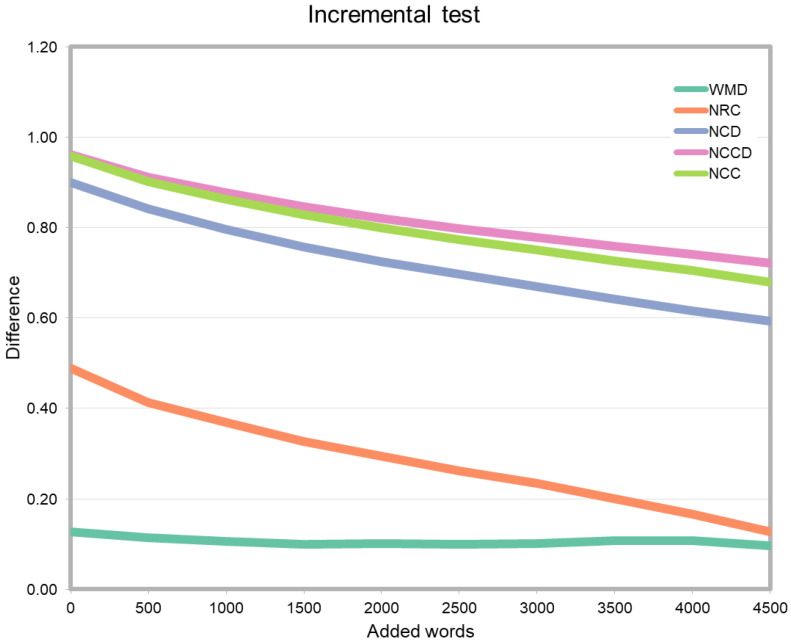
Incremental test to evaluate accuracy. We compare *file1* with *file2*, then, we incrementally add words from the second file to the first one. As expected, the difference softly decays when measuring NRC. On the other hand, WMD does not behave as expected, and the difference remains flat, and in some cases, it even grows. NCD, NCCD, and NCC, also behave well in this test.

Estimating the differences between two scientific texts is difficult to describe formally. Previous literature has used citations ([27]), document contents ([28]) or keyword analysis ([1]). However, there is not a universally accepted baseline that helps us properly analyzing the accuracy of the different measures for scientific papers comparison. We can, despite that, measure accuracy in an indirect way. Given two different research papers, independently on how similar or different they are, there is a way to make them closer to each other: by incrementally taking words of the second paper and appending them to the first one, their difference should monotonically decrease. Thus, our first test compares the different measures using this strategy. We take two different files *X* and *Y* (properly processed as detailed above), and execute a program that fetches 500 words from *Y* and appends them to *X*. Then, a second run appends 500 more, and so on. Finally, we measure the differences between the different versions of the files. As expected, the results should show that the difference value decreases softly as the number of words shared by the two papers grows. The results are shown in Figure 2. As can be seen, for WMD, although the shape of the curve indicates some decrement, it is almost flat and even grows in some points. NRC, NCD, NCCD, and NCC, on the other hand, behave as expected.

In light of the results, we can discard WMD for our problem, and the next tests will show only NRC, NCCD, NCC, and NCD.

#### 4.4.2. Author Style vs. Contents

Previous literature has shown that NRC can be used for text attribution. More concretely, Pinho et al. [10] successfully used NRC to assign texts to authors from a corpus of 168 books. They demonstrated that their system, which uses a specifically developed compressor to perform relative compression, was able to properly classify all the texts in the corpus with no error. In light of this, we asked ourselves whether using NRC on our processed corpus of scientific papers would be measuring author style or document contents. Please note that since our text processing pipeline includes stop word removal and stemming, we expect the author style to be partially stripped from the resulting text, and thus NRC to work as expected.

To ensure that our system was going beyond author style classification, we performed the following experiment: We took several visualization papers of different authors on volume rendering. The papers belong to the authors’ initial research, since they are published during the PhD thesis development. Therefore, they have lots in common (typically a coarse research field), and most likely, are going to have the author’s own writing style. We process the papers with the aforementioned pipeline, and calculate the NRC among all of them. Then, we evaluate the differences (in both directions) among the papers.

**Figure 3:**
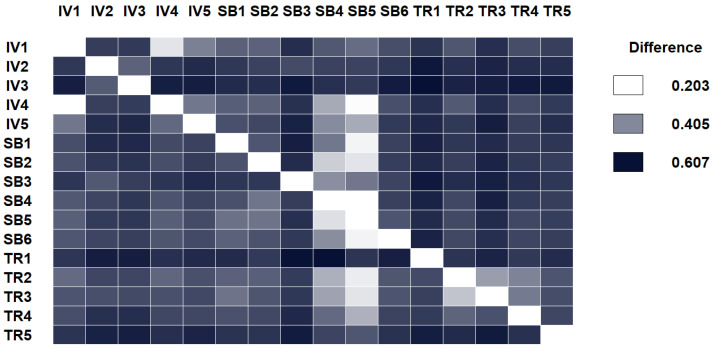
Comparing papers of three different authors to evaluate whether NRC goes beyond author style. Papers were selected among the initial papers of three researchers: Dr Stefan Bruckner, Dr Timo Ropinski, and Dr Ivan Viola. The first and the last one made their PhD thesis in the same institution, and similar area, so one might expect similar writing style. However, smaller differences do not lie among the authors of the same authorship or institution.

If NRC only accounts for authorship attribution after our text processing, it will issue smaller values of difference for the papers of the same author. Otherwise, the distances will correspond more likely to the contents of the paper than their authors. Figure 3 shows the result of this evaluation. 15 papers from three different authors were analyzed: Stefan Bruckner, Timo Ropinski, and Ivan Viola (the full list is in the Appendix A and Appendix B).

It turns out that comparing the papers using NRC on our dataset does not find smaller differences papers based on its author (or for authors belonging to the same institution), but more in line with its contents. Of course, some papers of the same author have very small differences because they are very closely related, and we cannot discard that the authorship is there, though at a lower proportion. However, other papers score higher than with respect to other authors. Please note that even a manual accurate selection on what two papers are closest to each other is, when papers are on a similar subject, very difficult to achieve. Though the style of an author may change from year to year, this test seems to confirm that NRC is able to approximate the level of novelty (and probably the inherent part of author evolution). Therefore, it seems that NRC, together with the data processing we performed, can effectively help us classify papers based on contents, not on authorship.

**Figure 4:**
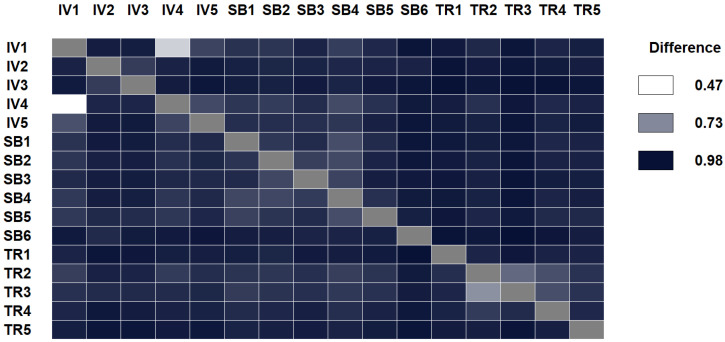
Comparing papers of three different authors to evaluate whether NCC goes beyond author style. As with NRC, smaller differences not always correspond to papers of the same author, although in this case, more comparisons seem to accumulate near the upper bound.

We also ran the same experiment using NCC and the results are shown in Figure 4. Like in the previous case, smaller differences seem not always come from the same authorship, but the differences here are more difficult to notice. NCCD yields similar values than NCC in this case, with no clear separation of authors, but, again, more values in the upper bound.

**Figure 5:**
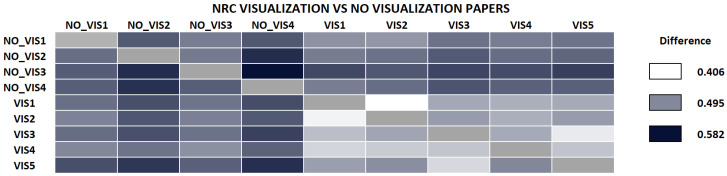
Comparison of papers from and outside the visualization research field. Note how all the calculated differences between visualization papers are consistently smaller than when we calculate the difference between a visualization paper and a paper that does not belong to the area. This indicates that NRC can classify research areas pretty well.

We performed a second test to see whether NRC was able to classify according to research areas. Here, we selected a small set of visualization papers (randomly chosen from the corpus of IEEE VisWeek), and downloaded a small random set of papers from different areas, mainly from *arxiv.org*. The complete list of the papers used in this experiment is also in the Appendix A and Appendix B. Intuitively, if we compare all papers against each other, the differences between visualization papers should be lower than those papers against the other ones. Figure 5 shows the results. Please note that the difference between visualization papers, is always smaller than when we compare visualization papers with papers of outside research fields (except for VIS1, and VIS2 with VIS5 in one direction).

**Figure 6:**
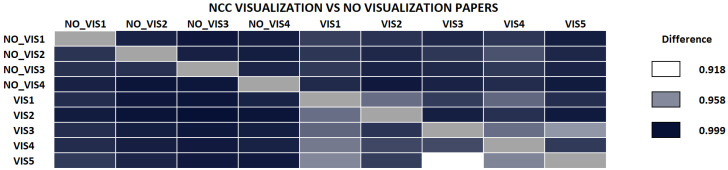
Comparison of papers from and outside the Visualization research field using Normalized Conditional Compression. In this case, some papers, such as VIS3, VIS4, and VIS5 exhibit smaller differences with non-visualization papers than with some visualization papers (e.g., VIS2).

We also performed the same comparison with NCC, shown in Figure 6. Compared to the previous case, though the measure separates well some of the visualization papers from other papers, some of them are not properly classified, such as VIS3, VIS4, and VIS5 papers. NCC value between VIS2 and VIS3 (see column VIS3) is larger than NCC between all non-visualization papers and VIS3. The same happens with VIS5 versus VIS2 in the same direction. We also tested the same dataset with NCD, and, though the difference values are sometimes smaller between visualization and non-visualization papers, the number of papers misclassified is larger than with NRC, NCCD, or NCC. In the following, we have mostly concentrated on the NRC, although NCC behaves similarly in most cases. When necessary, we note the differences.

#### 4.4.3. Containment

The goal of this test is to evaluate whether NRC can be used to estimate the novelty of a paper with respect to the other. In this case, we take advantage of fact that NRC is a semi-distance. Again, finding a baseline is complex, so we opted for the two following experiments: First, we compare what happens if we compare a State-of-the Art paper with respect to a set of papers of the same area, and then we compare a paper with a follow-up of the same authors. The intuition says that the difference from the STAR to another paper will be smaller than the difference from a paper of the same area to the STAR, since, in some sense, the STAR describes a larger amount of concepts, and therefore *contains* the papers in the area.

**Figure 7:**
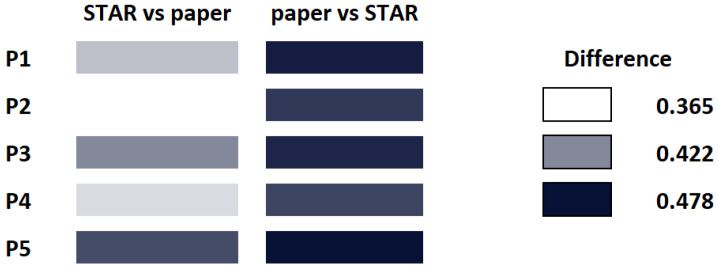
Comparing a STAR of molecular visualization with a set of papers of the same area. Notice how the difference of each individual paper with respect to the STAR is larger than the difference of the STAR paper with respect to the other papers. This seems to indicate that the STAR somewhat *contains*, at least partially, the contents of the papers.

For this comparison, we downloaded a STAR on molecular visualization, and several papers of the same field. The results can be seen in Figure 7. As we expected, the difference from the STAR to the papers is smaller than from the papers to the STAR, in all cases. NCC also passes this test. Like in previous cases, the concrete papers are enumerated in the Appendix A and Appendix B.

We did a second test of a related concept: the follow-up paper. It is similar than the previous, but in this case, the two are more closely related. Intuitively, a follow-up paper will also *contain* the initial paper. Again, the testing with two papers that can be ensured that one is the follow-up of the other yield the same results, as shown in Table 1. NCC can also be evaluated with this test, and as in the previous case, it also passes it.

#### 4.4.4. Validation Results

All these tests prove that NRC, combined with our text processing, is a powerful tool for document comparison, and that can be successfully used for the visualization of research evolution of a person or a conference. NCC is close to NRC, as it also passes the containment test, but does not classify papers as clearly as NRC. To sum up, the different tests are depicted in Table 2. Although WMD was discarded in our accuracy test, because it does not show a monotonic behavior, we also tested it less formally for the other tasks.

Please note that the problem we dubbed *containment* benefits from the fact that NRC is a semi-distance. Since NCD, NCCD, and WMD are symmetric, these cannot be used to estimate this property.

## 5. Results

Now we proceed to point how the NRC can be used to visualize documents collections or author profiles with the goal of visually explaining their evolution. For all the experiments, we used the relative compressor by Pinho et al. [10].

### 5.1. Evaluating Conference Evolution

One application of our technique is to evaluate how a conference evolves through the years. We can use it to have some insights on whether the developments are more incremental or there are great differences (that may indicate innovative contents). We can get some idea by analyzing how the different papers in one year compare to another, and how the whole conference edition compares to another one.

**Figure 8:**
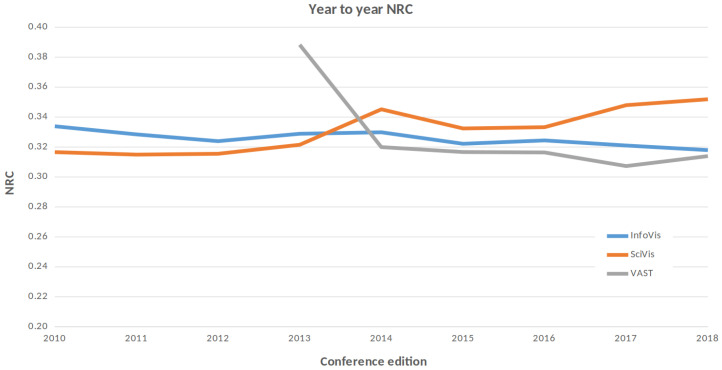
The evolution of the three conferences of the VisWeek for the last ten years. Please note that VAST only publishes in TVCG since 2009, so we compare the evolution year to year since the first year we have information.

For comparing the degree of difference between one year and another, we take all the papers of a conference edition (properly processed), and append them in a single file. By doing the same for each edition, we can compare editions of a conference, as well as differences between one conference and another. Figure 8 shows the evolution of the three conferences of the IEEE VisWeek year to year following this strategy. In this case, we compare each conference with the previous edition. The charts seem to indicate that their degree of change has decreased slowly in the case of InfoVis, while the SciVis has increased a little bit recent years.

**Figure 9:**
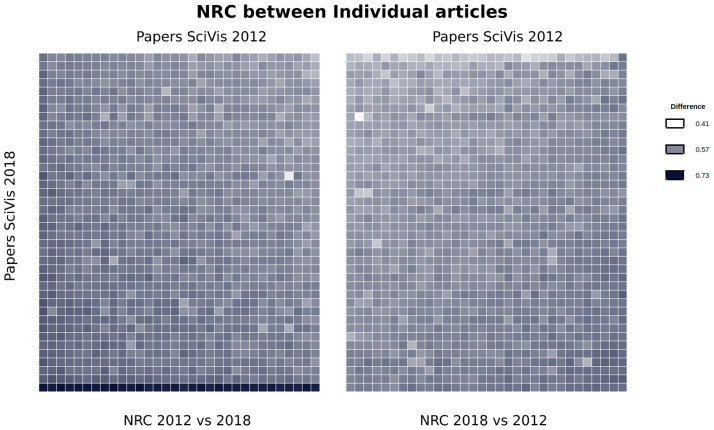
Comparison of the papers of SciVis 2012 vs. SciVis 2018. The heatmap has been sorted so that the darker colors are in the bottom left and bottom right of the rectangles, respectively. To keep symmetry, the conference editions are placed in the same position. The left map contains the comparison NRC(SciVis2010, SciVis2018), while the right map contains the evaluation of NRC(SciVis2018, 2010). So, the left one would indicate how different are the papers from the second conference with respect to the ones of the first one. This visualization allows a quick gaining of certain insides. For example, the bright white spot on the left heatmap corresponds to two papers on Morse–Smale complexes published in 2012 and 2018 by the same authors.

A second test that can be done is by comparing all the papers against each other, from one edition to the other. This might highlight some paper that is clearly different from the previous ones. We did this in Figure 9, where the 2012 and 2018 editions are compared. In this case, we measure the NRC between all the papers in both directions and display the information in a heat map. To compare the two conferences, we put the 2012 vs. 2018 comparison on the left, and the 2018 vs. 2012 on the right. Moreover, to keep the symmetry and facilitate reading, we sort the heatmap matrix in the same order (SciVis 2018 papers are in rows and SciVis 2012 papers are columns).

To facilitate the interpretation, we sort the rows and columns so that larger values are moved to the bottom left and bottom right borders, respectively. This facilitates the interpretation by: i) it is easy to see whether one of the conference editions is better at *predicting* the other: lighter colors indicate the ability of an edition to predict the other. Note how the 2018 vs. 2012 comparison (right) shows lighter colors than the 2012 to 2018, which may give a hint on the fact that the 2018 edition has novel contributions with respect to the 2012 edition.

A second advantage of this layout is that we can easily identify papers that seem highly innovative, or, at least different, such as the bottom row of left map. This is the case for the 2018 paper entitled “Hexahedral Mesh Structure Visualization and Evaluation”, which has large distances from any of the papers on the 2012 edition. This may be an indication that the paper has something that places it apart from the others. With this color coding and data sorting, it is very simple to identify such cases. Another clear case is the bright spot in the left heatmap. This corresponds to two different papers (in 2012 and 2018) that deal with Morse–Smale complexes which actually share the same authors. This kind of information would be very difficult to easily detect by other means.

### 5.2. Author Profile Analysis

Presently, with Visualization and Computer Graphics evolving so fast, researchers may sometimes change their research focus. Consequently, it may be interesting to see whether an author has a publication profile within a single area, or, on the contrary, she changes the research area along her career. One way to do so is to compare the papers published along several years, and the differences between the initial one(s) and the rest. This is what we did for a certain researcher. In this case, we took the papers published from 2003 and 2007 by Dr Ivan Viola, and they belong to the volume rendering area. We compare them in two different ways: first, we compare all the papers with the first one (in 2003), and second, we compare the papers year to year. Please note that as described in the Appendix A and Appendix B, the first paper we took was the first one where he was first author. Then, for all the papers from 2004 and later, the difference is computed as:

(5) $$DIFF{\left(i\right)}_{y}=NRC\left(Paper_{2003},Paper_{i,y}\right),$$

where $Paper_{2003}$ is the reference paper, and $Paper_{i,y}$ is the paper number *i* paper of year *y*. This means that we calculate how different is the new paper with respect to the older one. The indices of the paper happen to be arranged according to download PDF names. The full list of the papers with the ids that appear in the chart are described in the Appendix A and Appendix B.

**Figure 10:**
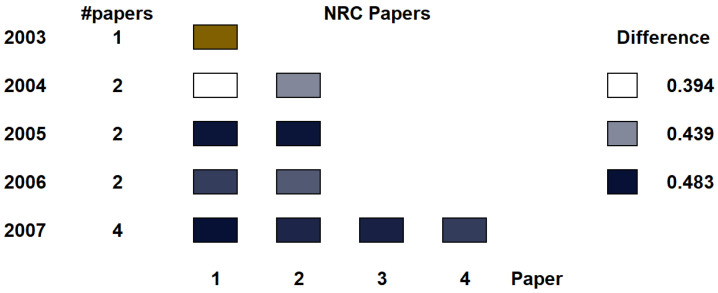
The figure shows the relative distances of the papers from the initial paper (in brown) from 2003.

In Figure 10 we show the difference of the papers with respect to the first in year 2003. The left column identifies the year, while the second column counts the number of papers for the year. The bottom row indicates the paper number within the year. For each paper, a heatmap encodes the difference calculated using NRC. For the heatmap, we use white for the minimum difference, and dark blue for the largest one.

If we analyze the resulting chart, the differences seem to indicate that his research field expands a little bit almost every year. Moreover, we can also extract another insight from the chart: it seems that the first paper in 2004 (second row, left) has high relationship with the paper in 2003. Indeed, the paper on 2003 is about hardware-accelerated filtering and segmentation of volume data, and the first paper in 2004 is about GPU-based frequency domain rendering, while the second one in 2004 is about importance-driven volume rendering.

**Figure 11:**
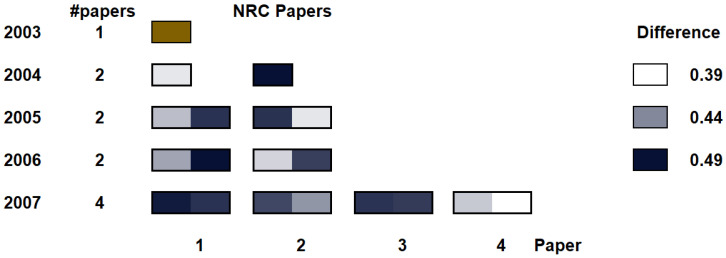
Comparison of the research papers from 2003 to 2007, year to year. In this case, each paper compares to the ones in the previous year, to analyze year-to-year evolution. The last paper of 2007, for instance is highly related to the second paper of 2006 (see the clear color), since both deal with importance-driven focus of attention in visualization.

We can also visualize the profile of the researcher in a different manner. We can see the year-to-year evolution, as shown in Figure 11. In this case, we compare each paper with the papers of the previous year, and this is depicted as an individual heatmap *per paper*. In this case, the evaluate the difference, for each paper *i* in year *y*, with all the papers *j* in year y-1, the following way:

(6) $$DIFF{\left(i,j\right)}_{y}=NRC\left(Paper_{j,y-1},Paper_{i,y}\right),$$

note that this means that the NRC is capturing the relative *novelty* of paper *i* with respect to the ones in the previous year, not the other way around.

Like in the previous case, the heatmap is colored using white for the minimum difference and a medium-dark blue for the largest difference value among all of them. We can see how some of the papers each year seem to be innovative with respect to the papers in the previous year, while some of them are more incremental. One may be curious about the last paper in 2007, which seems to have a low difference with the second paper of 2006. It turns out that the 2006 paper is about importance-driven focus of attention, and the 2007 paper is about the role of topology in focus+context visualization. Therefore, this seems to indicate that NRC is making a good job at evaluating researcher evolution.

This chart will naturally require more horizontal space than the previous one, since the number of papers that an author publishes typically grows with the time. However, this value also saturates around a dozen or less (most well-known profiles in the visualization area actually have smaller numbers, although some may grow up to doubling this size). Therefore, since heatmaps can be very compact, we believe the depiction is valid for a wide range of situations.

### 5.3. Details and Performance

All the experiments have been performed using the conditional compressor by Pinho et al. [10], which kindly provided us with the code. The compressor can deal with regular compression, conditional compression, and relative compression. This ensures the fairness with respect to timings and compression ratio.

To perform the compression, we used the compressor with context models of order 2 (α=1/10), 3 (α=1/100), 5 (α=1/500), and 7 (α=1/1000), for both the reference and the target files where required. The configuration was found empirically after testing parameters ranging from 2 until 9, and alphas going from 1/10 till 1/10000. Larger orders result in a slightly slower compression time but did not significantly (if at all) improve the result in our case.

As shown, NRC outperforms NCD in different areas, such as separating research areas. NCC also behaves better than NCD and the other measures tested in these experiments. Moreover, computing NCD requires three compressions (*x*, *y*, and xy), which makes the computation time around three times the cost of calculating NRC(x,y) with the same compressor.

Concerning NCCD, it achieves similar results than NRC, although we found that they seem to be slightly worse in the research field classification. One reason to choose NCCD over NRC would be the unavailability of a conditional compressor. Sculley and Brodley [37], as already mentioned, use the approximation C(x|y)=C(yx)-C(y), which can be computed using a regular compressor. Unfortunately, this still means to compress the sequences four times (for *x*, *y*, xy, and xy). Consequently, the time required to compute NCCD with the same compressor used for NRC is roughly four times larger (NRC took around 0.4408s per evaluation versus 0.11275s of NRC). Please note that if the compressor used is a *regular* compressor as defined in Cilibrasi and Vitányi [11,38], C(xy)=C(yx), so the cost could be reduced to roughly three times slower.

Finally, NCC achieves similar results to NRC, though it seems that it can separate research areas less effectively than NRC. Concerning the cost, in our experiments, NCC required 0.3305 seconds per evaluation on average over several files of different sizes, while NRC requires 0.11275 seconds with the same parameters and same file set. However, as mentioned above, NCC could be computed using the approximation C(x|y)=C(yx)-C(y), which would allow us to use a regular compressor. For instance, *7za*, a compressor that performs Prediction by Partial Matching and is especially designed for speed, achieves a much faster compression timings that the compressor used here. However, since the one used here was not designed for speed, doing a numerical comparison would be unfair. Other tools for compression could be used [38].

## 6. Discussion

In this paper, we have presented a data-processing pipeline and a method to use Normalized Relative Compression to estimate the difference between research papers. We have also introduced how to use well-known visualization techniques to express those differences to get insights on how a certain conference evolves and how the profile of an author changes along the time. The developed pipeline has been validated through a set of experiments that demonstrate that NRC can be used to investigate how a certain research area fares, as well as to visually depict author profiles.

We used several series of papers, and the largest dataset was from the last 10 years of IEEE VisWeek conference. In the future, we would like to investigate with broader sets of data, such as downloading data from other conferences, as well as other researcher profiles. Currently, our system only uses text, but an obvious extension would be to include images. In this case, not only the image processing, but the image extraction alone, presents important challenges to do it in a fully automatic fashion.

## Figures and Tables

**Table 1:** Comparing a paper with a follow-up. As expected, the NRC between the original paper and the follow-up is larger than in the other direction.

	Initial Paper	Follow-up
**Initial paper**		0.439
**Follow-up**	0.408	

**Table 2:** Summary of the requirements, and their fulfillment by the different difference measures. Accuracy has been measured throughout the *incremental* test: two files that are increasingly similar should yield monotonically decreasing differences/distances. The third column refers to the ability of finding similarities between research areas. Please note that for all the measures tested, only NRC fulfills all the desired properties. NCC behaves quite similarly to NRC, but seems slightly worse in research areas classification, so more testing is probably needed. NCCD can separate research areas up to a certain extent, as showed above, but less clearly than NRC. With respect to the *containment*, only NRC and NCC can detect that a file *contains* the other.

Technique	Accuracy Test	Separates Research Areas	Contents over Style	Containment
NRC	✓	✓	✓	✓
NCC	✓	(✓)	(✓)	✓
NCCD	✓	✗	✗	N/A
NCD	✓	✗	✗	N/A
WMD	✗	(✓)	(✓)	N/A

## Appendix A. Documents Used in the Experiments

We needed to select sets of articles for the different experiments. Please note that these papers were not cherry-picked. We only searched for researchers or papers that we were aware they did belong to the same area (such as for the STAR test, or the volume rendering set of papers). Once decided the sources of information, we downloaded the files that were available through PDFs. For example, the most of the non-visualization papers were selected by choosing different research fields in the *recents* part of *arxiv.org*, and taking the one of the first papers that appeared there.

### Appendix A.1. Paper Classification Experiment

For the analysis of author versus style, we used the following papers:

IV1 Viola, I., Kanitsar, A., & Groller, M. E. (2005). Importance-driven feature enhancement in volume visualization. IEEE Transactions on Visualization and Computer Graphics, 11(4), 408–418.
IV2 Viola, I., Kanitsar, A., & Gröller, M. E. (2004, April). GPU-based frequency domain volume rendering. In Proceedings of the 20th spring conference on Computer graphics (pp. 55–64). ACM.
IV3 Viola, I., Kanitsar, A., & Groller, M. E. (2003, October). Hardware-based nonlinear filtering and segmentation using high-level shading languages. In Proceedings of the 14th IEEE Visualization 2003 (VIS’03) (p. 41). IEEE Computer Society.
IV4 Viola, I., Kanitsar, A., & Groller, M. E. (2004, October). Importance-driven volume rendering. In Proceedings of the conference on Visualization’04 (pp. 139–146). IEEE Computer Society.
IV5 Viola, I., & Gröller, M. E. (2005, May). Smart visibility in visualization. In Computational Aesthetics (pp. 209–216).
SB1 Bruckner, S., & Groller, M. E. (2006). Exploded views for volume data. IEEE Transactions on Visualization and Computer Graphics, 12(5), 1077–1084.
SB2 Bruckner, S., & Gröller, E. (2007). Enhancing depth-perception with flexible volumetric halos. IEEE Transactions on Visualization and Computer Graphics, 13(6), 1344–1351.
SB3 Bruckner, S., & Gröller, M. E. (2007, September). Style transfer functions for illustrative volume rendering. In Computer Graphics Forum (Vol. 26, No. 3, pp. 715–724). Oxford, UK: Blackwell Publishing Ltd.
SB4 Bruckner, S., & Groller, M. E. (2005). Volumeshop: An interactive system for direct volume illustration (pp. 671–678). IEEE.
SB5 Grimm, S., Bruckner, S., Kanitsar, A., & Gröller, E. (2004). Flexible direct multi-volume rendering in dynamic scenes. In VMV (pp. 379–386).
SB6 Rautek, P., Csébfalvi, B., Grimm, S., Bruckner, S., & Gröller, M. E. (2006, May). D 2 VR: high-quality volume rendering of projection-based volumetric data. In Proceedings of the Eighth Joint Eurographics/IEEE VGTC conference on Visualization (pp. 211–218). Eurographics Association.
TR1 Ropinski, T., Steinicke, F., & Hinrichs, K. (2006, July). Visually supporting depth perception in angiography imaging. In International Symposium on Smart Graphics (pp. 93–104). Springer, Berlin, Heidelberg.
TR2 Ropinski, T., Steinicke, F., & Hinrichs, K. H. (2005). Interactive importance-driven visualization techniques for medical volume data. In Proceedings of the 10th International Fall Workshop on Vision, Modeling, and Visualization (VMV05) (pp. 273–280).
TR3 Ropinski, T., Steinicke, F., & Hinrichs, K. (2005). An Efficient Approach for Emphasizing Regions of Interest in Ray-Casting-based Volume Rendering.
TR4 Ropinski, T., Steinicke, F., & Hinrichs, K. (2006). Visual exploration of seismic volume datasets.
TR5 Ropinski, T., Specht, M., Meyer-Spradow, J., Hinrichs, K. H., & Preim, B. (2007, November). Surface glyphs for visualizing multimodal volume data. In VMV (pp. 3–12).

We used the following papers in the experiment that separates visualization papers from other research areas. The papers were picked from *arxiv.org* and other sources, and some random picks from the IEEE VisWeek corpus:

NO_VIS1 Deshpande, A., & Ouldridge, T. E. (2019). Optimizing enzymatic catalysts for rapid turnover of substrates with low enzyme sequestration. arXiv preprint arXiv:1905.00555.
NO_VIS2 Morrill, G., Kuznetsov, S., Kanovich, M., & Scedrov, A. (2018, August). Bracket induction for Lambek calculus with bracket modalities. In International Conference on Formal Grammar (pp. 84–101). Springer, Berlin, Heidelberg.
NO_VIS3 Liu, S. (2019). Compactification of Extensive Forms and Belief in the Opponents’ Future Rationality. arXiv preprint arXiv:1905.00355.
NO_VIS4 Liu, B., Lai, D., & Wang, Y. H. (2019). Black Hole and Neutron Star Binary Mergers in Triple Systems: II. Merger Eccentricity and Spin-Orbit Misalignment. arXiv preprint arXiv:1905.00427.
VIS1 Xu, K., Xia, M., Mu, X., Wang, Y., & Cao, N. (2019). EnsembleLens: Ensemble-based Visual Exploration of Anomaly Detection Algorithms with Multidimensional Data. IEEE transactions on visualization and computer graphics, 25(1), 109–119.
VIS2 Chen, W., Guo, F., Han, D., Pan, J., Nie, X., Xia, J., & Zhang, X. (2019). Structure-Based Suggestive Exploration: A New Approach for Effective Exploration of Large Networks. IEEE transactions on visualization and computer graphics, 25(1), 555–565.
VIS3 Ament, M., Weiskopf, D., & Carr, H. (2010). Direct interval volume visualization. IEEE transactions on visualization and computer graphics, 16(6), 1505–1514.
VIS4 Ankele, M., & Schultz, T. (2019). DT-MRI streamsurfaces revisited. IEEE transactions on visualization and computer graphics, 25(1), 1112–1121.
VIS5 Lee, B., Yun, J., Seo, J., Shim, B., Shin, Y. G., & Kim, B. (2010). Fast high-quality volume ray casting with virtual samplings. IEEE Transactions on visualization and computer graphics, 16(6), 1525–1532.

### Appendix A.2. Author Profile Evolution Test

For the evolution of an author, we selected Assoc. Prof Ivan Viola. Since he has some papers with other authors at the beginning of his research career, and we felt that we needed to compare with the initial papers of his PhD thesis, we started in 2003. The list of papers is the following one:

2003 Viola, I., Kanitsar, A., & Groller, M. E. (2003, October). Hardware-based nonlinear filtering and segmentation using high-level shading languages. In Proceedings of the 14th IEEE Visualization 2003 (VIS’03) (p. 41). IEEE Computer Society.
2004,1 Viola, I., Kanitsar, A., & Gröller, M. E. (2004, April). GPU-based frequency domain volume rendering. In Proceedings of the 20th spring conference on Computer graphics (pp. 55–64). ACM.
2004,2 Viola, I., Kanitsar, A., & Groller, M. E. (2004, October). Importance-driven volume rendering. In Proceedings of the conference on Visualization’04 (pp. 139–146). IEEE Computer Society.
2005,1 Artner, M., Möller, T., Viola, I., & Gröller, M. E. (2005, June). High-quality volume rendering with resampling in the frequency domain. In EuroVis (pp. 85–92).
2005,2 Viola, I., & Gröller, M. E. (2005, May). Smart visibility in visualization. In Computational Aesthetics (pp. 209–216).
2006,1 Rautek, P., Viola, I., & Groller, M. E. (2006). Caricaturistic visualization. IEEE Transactions on Visualization and Computer Graphics, 12(5), 1085–1092.
2006,2 Viola, I., Feixas, M., Sbert, M., & Groller, M. E. (2006). Importance-driven focus of attention. IEEE Transactions on Visualization and Computer Graphics, 12(5), 933–940.
2007,1 Balabanian, J. P., Viola, I., Ona, E., Patel, R., & Gröller, M. E. (2007, May). Sonar Explorer: A New Tool for Visualization of Fish Schools from 3D Sonar Data. In EuroVis (pp. 155–162).
2007,2 Burns, M., Haidacher, M., Wein, W., Viola, I., & Gröller, M. E. (2007, May). Feature emphasis and contextual cutaways for multimodal medical visualization. In EuroVis (Vol. 7, pp. 275–282).
2007,3 Tóth, Z., Viola, I., Ferko, A., & Gröller, E. (2007). N-dimensional Data-Dependent Reconstruction Using Topological Changes. In Topology-based Methods in Visualization (pp. 183–198). Springer, Berlin, Heidelberg.
2007,4 Viola, I., & Gröller, E. (2007). On the role of topology in focus+ context visualization. In Topology-based Methods in Visualization (pp. 171–181). Springer, Berlin, Heidelberg.

### Appendix A.3. Containment Experiments

In this experiment, we compared a State-of-the-Art paper on molecular visualization with different papers of different researchers. Again, the papers were not cherry-picked. We visited web pages of different researchers working in the area, and chose one paper from the field from their publication list.

STAR Kozlikova, B., Krone, M., Falk, M., Lindow, N., Baaden, M., Baum, D., Viola, I., Parulek, J. & Hege, H. C. (2015). Visualization of biomolecular structures: State of the art.
P1 Kottravel, S., Falk, M., Sundén, E., & Ropinski, T. (2015, April). Coverage-based opacity estimation for interactive depth of field in molecular visualization. In 2015 IEEE Pacific Visualization Symposium (PacificVis) (pp. 255–262). IEEE.
P2 Grottel, S., Krone, M., Scharnowski, K., & Ertl, T. (2012, February). Object-space ambient occlusion for molecular dynamics. In 2012 IEEE Pacific Visualization Symposium (pp. 209–216). IEEE.
P3 Tarini, M., Cignoni, P., & Montani, C. (2006). Ambient occlusion and edge cueing for enhancing real time molecular visualization. IEEE transactions on visualization and computer graphics, 12(5), 1237–1244.
P4 Duran, D., Hermosilla, P., Ropinski, T., Kozlikova, B., Vinacua, A., & Vázquez, P. P. (2019). Visualization of large molecular trajectories. IEEE transactions on visualization and computer graphics, 25(1), 987–996.
P5 Lindow, N., Baum, D., & Hege, H. C. (2018). Atomic accessibility radii for molecular dynamics analysis.

For the second experiment, which was basically a proof-of-concept, we selected a paper that we knew surely had a follow-up, and compared both. In this case, the easiest to do was to use papers from the author of this article. The selected papers were:

Initial Vázquez, P. P., Feixas, M., Sbert, M., & Heidrich, W. (2001, November). Viewpoint selection using viewpoint entropy. In VMV (Vol. 1, pp. 273–280).
Follow-up Vázquez, P. P., Feixas, M., Sbert, M., & Llobet, A. (2002, May). Viewpoint entropy: a new tool for obtaining good views of molecules. In ACM International Conference Proceeding Series (Vol. 22, pp. 183–188).

## Appendix B. Acronyms

Several acronyms were used along the text. Here is a list of all of them:

EMD: Earth Mover’s Distance. A distance metric between two probability distributions. In mathematics it is known as the Wasserstein metric.
InfoVis: IEEE Information Visualization, the international conference on information visualization, organized yearly by IEEE in the so-called VisWeek, along with SciVis and VAST.
NCCD: Normalized Conditional Compression Distance.
NCD: Normalized Compression Distance.
NRC: Normalized Relative Compression.
TVCG: IEEE Transactions on Visualization and Computer Graphics, the IEEE journal on visualization.
SciVis: IEEE Scientific Visualization, the international conference on scientific visualization, organized yearly by IEEE in the so-called VisWeek, along with InfoVis and VAST.
VAST: IEEE Visualization Analytics Science and Technology, the international conference on visual analytics, organized yearly by IEEE in the so-called VisWeek, along with InfoVis and SciVis.
WMD: Word Mover’s Distance. A distance metric based on deep learning.
